# Supplementary material for: Location is a major barrier for transferring US fossil fuel employment to green jobs
Source: Nat Commun. 2023 Sep 26;14:5711. doi: 10.1038/s41467-023-41133-9 (PMC10522673; doi:10.1038/s41467-023-41133-9)
Supplement: Supplementary file 1 — Supplementary Information [file 41467_2023_41133_MOESM1_ESM.pdf]

Supplementary Materials: Location is a major barrier for transferring US fossil fuel employment to green jobs

Junghyun Lim[1], Michaël Aklin[2], and Morgan R. Frank[3,4,5,*]

[1] *Perry World House, University of Pennsylvania, Philadelphia, PA 19104 USA*
[2] *Department of Political Science, University of Pittsburgh, Pittsburgh, PA 15216 USA*
[3] *Department of Informatics and Networked Systems, University of Pittsburgh, Pittsburgh, PA 15216 USA*
[4] *Digital Economy Lab, Institute for Human-Centered Artificial Intelligence, Stanford University, Stanford, CA 94305 USA*
[5] *Media Laboratory, Massachusetts Institute of Technology, Cambridge, MA, 02139 USA*
[*] *To whom correspondence should be addressed. E-mail: mrfrank@pitt.edu*

August 15, 2023

Contents

1 Labor Data for Fossil Fuel Workers **2**
1.1 Identifying Fossil Fuel Workers 2
1.2 Alternative Measure of Fossil Fuel Workers 2

2 Estimating Green Employment **3**
2.1 Identifying Green Occupations 4
2.2 Comparison with other measures of green occupations 6
2.2.1 Validating prediction for green job supply in 2029 6
2.2.2 Comparison: Princeton Net-Zero America Project 7

3 Skill similarities between industries and occupations **9**
3.1 Important skills for fossil-fuel workers 9
3.2 Important skills for green jobs 12
3.3 Skill similarities across industries 14

4 Modeling Fossil Fuel Workers Transitions to Other Industries **16**
4.1 Transition under different scenarios 18
4.2 Transition to non-green sectors 18
4.3 Transition to green jobs under different jobs supply scenario 19
4.4 Transition to different types of green jobs 20

5 Fossil Fuel Workers and Green Energy Plants **21**

1 Labor Data for Fossil Fuel Workers

1.1 Identifying Fossil Fuel Workers

SOC	Occupation Title
47-5011	Derrick Operators, Oil and Gas
47-5012	Rotary Drill Operators, Oil and Gas
47-5013	Service Unit Operators, Oil and Gas
47-5022	Excavating and Loading Machine/ Dragline Operators, Surface Mining
47-5041	Continuous Mining Machine Operators
47-5043	Roof Bolters, Mining
47-5044	Loading and Moving Machine Operators, Underground Mining
47-5071	Roustabouts, Oil and Gas
47-5081	Helpers–Extraction Workers

SI Table 1: List of occupations in fossil-fuel extraction.

This section defines how we measure the number of fossil fuel workers in metro and non-metro areas. We discuss alternative definitions below.

The Bureau of Labor Statistics (BLS) collects employment data for over 750 occupations, 232 of which are present in the fossil fuel industry. Among these, we focus on 11 occupations that fall into the "extraction workers" category (Table 1). We focus on these occupations for two reasons. First, they are well represented in the fossil fuel sector: about 70% of extraction workers are active in fossil fuel firms (as of 2019). Second, they represent a fundamental core of the fossil fuel business (representing about 27% of the workers in the industry) and their livelihood depends the most fundamentally on the fossil fuel sector. The next largest groups within fossil fuel firms – engineers and material movers – represent much smaller shares (about 13% and 10% respectively) of fossil fuel workers and have employment opportunities outside of fossil fuel related firms. As such, we use a narrow definition of fossil fuel workers in order to focus on the segment that is the most at risk in the clean energy transition.

We collect the number of workers of extraction occupations in metro and non-metropolitan areas where fossil fuel is extracted. In other words, we exclude workers of extraction occupations who work in areas without any fossil fuel extraction. To identify areas with active fossil fuel extraction activities, we use county-level oil, gas, and coal production data sourced from the U.S. Department of Agriculture [1]. See Figure 1A for the spatial distribution of extraction workers across the US.

For comparison, we show the spatial distribution of the entire workforce of fossil-fuel industry using data from the Business Dynamics Survey (BDS) from the US Census Bureau. BDS provides the number of workers in metropolitan areas at the industry level, following the North American Industry Classification System (NAICS) two-digit code (Figure 1B).

1.2 Alternative Measure of Fossil Fuel Workers

In this section, we replicate our main findings using alternative ways to measure the number of fossil fuel workers. While we focus on extraction workers as the core workforce in the fossil fuel industry, there are broader sets of occupations present in the fossil fuel industry. As an alternative, here, we focus on 40 different occupations present within the fossil fuel industry that require manual labor as their main tasks (i.e., manual workers). This criterion includes all the extraction occupations and other occupations in "Installation, Maintenance, and Repair Occupations (SOC code 49-0000)", "Production Occupations (SOC code 51-000)", and some of the occupations in "Transportation and Material Moving Occupations (53-0000)."

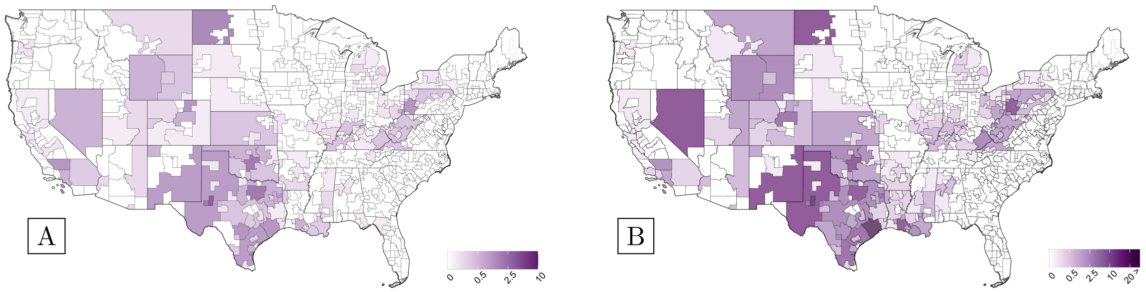

SI Figure 1: **(A)** The distribution of fossil fuel extraction workers across metropolitan and non-metropolitan areas in 2019 (i.e., major occupation group SOC 47-5000). **(B)** The distribution of the entire fossil-fuel workforce. The size of the fossil fuel workforce is measured based on the Business Dynamic Survey from the Census, which report the number of workers by sector following the NAICS taxonomy. We use the number of workers in "mining, quarrying, and oil and gas extraction (21)" sector in areas where oil, gas or coal productions are present. Unit of the workers are one thousand. Maps were made using the sf package in R (Pebesma E (2018). "Simple Features for R: Standardized Support for Spatial Vector Data." The R Journal, 10(1), 439–446.–CC-BY Attribution 4.0).

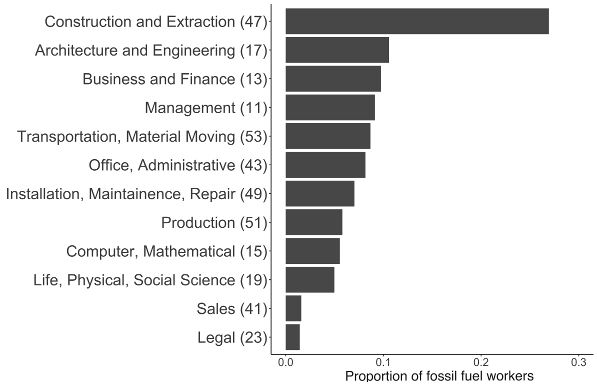

SI Figure 2: Proportion of fossil fuel workers by occupation group. The occupation groups are defined following the 2-digit Standard Occupation Classification system.

We use the number of workers with manual occupations within fossil fuel industry in metro and non-metropolitan areas where fossil fuel is extracted, and use them as a proxy for the number of fossil fuel workers. In other words, we exclude workers with manual occupations but work in areas without any fossil fuel production. Using the number of manual workers as input, we predict the number of manual workers who will be able to transition to green jobs. Using this alternative criterion for fossil fuel workers, we get consistent results similar to the analysis in the main text: The proportion of fossil fuel workers who transition to green jobs in 15 most fossil fuel labor-intensive regions remains lower than 1.1% and the proportion of entire fossil fuel workers who transition to green occupation is about 0.78% (Figure 3).

2 Estimating Green Employment

This section explains how we generated estimates of green jobs. We also summarize the results of robustness tests in which we modify our measurement strategy.

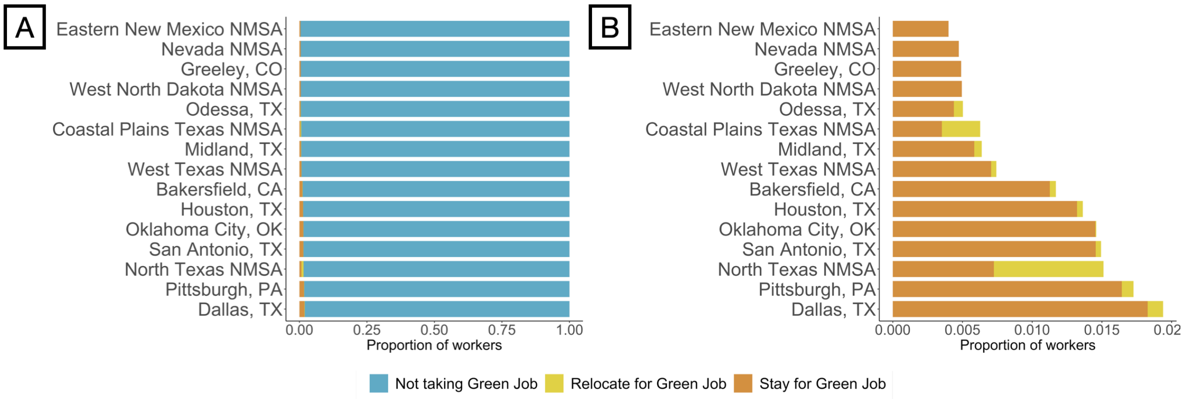

SI Figure 3: **(A)** Proportion of fossil fuel who transition to green jobs in 15 most fossil fuel labor-intensive regions remains lower than 1.15 % when we focus on all the manual labor workers in the fossil fuel industry instead of focusing on extraction workers. **(B)** Within the workers who transition to green jobs, the share of workers who relocate for new green jobs remains low as well.

2.1 Identifying Green Occupations

To identify green occupations, we rely on the classification established by Dierdorff and colleagues [2], a measure that has since been used widely, including in the European Union [3] and the US Bureau of Labor Statistics. We use the list of green occupations provided by Dierdorff et al., and match these occupations with the standardized occupation code following the SOC 6-digit code.

Dierdorff and colleagues [2] identify green jobs based on (1) types and (2) the sub-sectors associated with each green job. First, green jobs are classified into three types: Green Increased Demand (GID), Green Enhanced Skills (GES), and Green New and Emerging (GNE). GID occupations are expected to see an increase in employment demand for existing occupations as green economy activities rise. GES occupations are also to experience increasing demand as a result of green economy activities, but the required tasks, skills and knowledge for each occupation may change in future. Lastly, GNE occupations are new types of jobs that can arise as the green economy develops. In our main analyses, we include all three categories of green jobs. Since we cannot observe potential future changes in the required skills for GES occupations, we measure their skill requirements using the skill requirements measured in 2019 release of the O*NET database produced by the US Bureau of Labor Statistics. This is based on the underlying assumption that the skill requirements in the future will remain similar to the current skill requirements for the same occupation.

Second, Dierdorff and colleagues [2] categorize the green occupations in the following 12 different green economy related sub-sectors. Some occupations belong to more than one sub-sector simultaneously.

- Renewable Energy Generation
- Energy Efficiency
- Energy Trading
- Research, Design, and Consulting
- Agriculture and Forestry
- Recycling and Waste Reduction
- Green Transportation
- Green Construction
- Energy and Carbon Capture
- Environment Protection
- Manufacturing
- Governmental and Regulatory

SOC	Type	Occupation Title
47-2231	GNE	Solar photovoltaic installers
49-9081	GNE	Wind turbine service technicians
49-9099	GNE	Installation, maintenance, and repair workers, all other
47-4099	GNE	Miscellaneous construction and related workers*
47-1011	GNE	First-line supervisors of construction trades and extraction workers
41-4011	GNE	Sales representatives, technical and scientific products*
47-2211	GES	Sheet metal workers*
49-9042	GES	Maintenance and Repair Workers, General
51-9012	GES	Separating, filtering.. and still machine setters, operators*
51-8099	GNE	Plant and system operators, all other
51-8013	GES	Power plant operators*
51-8012	GID	Power distributors and dispatchers
51-8011	GES	Nuclear power reactor operators
51-4041	GES	Machinists
19-4041	GES	Geological and hydrologic technicians*
19-4051	GES	Nuclear technicians
17-2051	GES	Civil engineers*
17-2071	GES	Electrical engineers*
17-2141	GES	Mechanical engineers*
17-2199	GNE	Engineers, all other*
11-3051	GNE	Industrial production managers*
11-3071	GES	Transportation, storage, and distribution managers*
11-9041	GNE	Architectural and engineering managers*
11-9199	GNE	Managers, all other*

SI Table 2: List of green occupations in Renewable Energy Sector. SOC reports the Standard Occupation Classification 6 digit code for each occupation. Occupations with $*$ are associated with different green sectors. For instance, Sheet metal workers (47-2211) are associated with green construction and manufacturing according to Dierdorff et al [2].

In the main analyses, we use the 24 occupations that are associated with the Renewable Energy Generation sector. The Renewable Energy Generation sector primarily includes occupations that are related to developing and using renewable energy sources including wind, solar, hydropower, hydrogen, biomass and geothermal.

We focus on Renewable Energy Generation sector occupations as core green occupations because occupations in Renewable Energy Generation are more closely associated with the green economy than to other sectors. One challenge of identifying green jobs is that many green occupations defined by Dierdorff are not exclusively confined to the green economy. For example, bus drivers are classified as GID occupations within the green transportation sector, which encompasses 'activities related to increasing efficiency and/or reducing environmental impact of various modes of transportation." While the demand for bus drivers is expected to increase as the green transportation sector expands, the proportion of green transportation employment within the overall employment of bus drivers may remain relatively small. Similar examples can be found in green manufacturing (i.e. 'Cutting, Punching and Press Machine Operators', 'Industrial Machinery Mechanics', 'Shipping, receiving and traffic clerks'). These examples indicate that there is a trade-off between using more inclusive criteria of green occupations and overestimation of potential employment opportunities in green occupations.

Renewable Energy Generation sector has a benefit of having occupations relatively more exclusive to renewable energy sector such as Solar photovoltaic installers, wind turbine technicians, or power plant operators in comparison to other sectors. [2] defines this sector as being "at the heart of most green discussions."

Despite the benefits of focusing on the Renewable Energy Generation occupations, it is important

to note that this definition may rule out some important green occupations and their growth. In order to address this concern, we compare different alternative strategies with our primary measure and show the high correlation between them (Figure 4), as well as examine the potential transition of fossil fuel workers to different green sectors in the SI section 4.4.

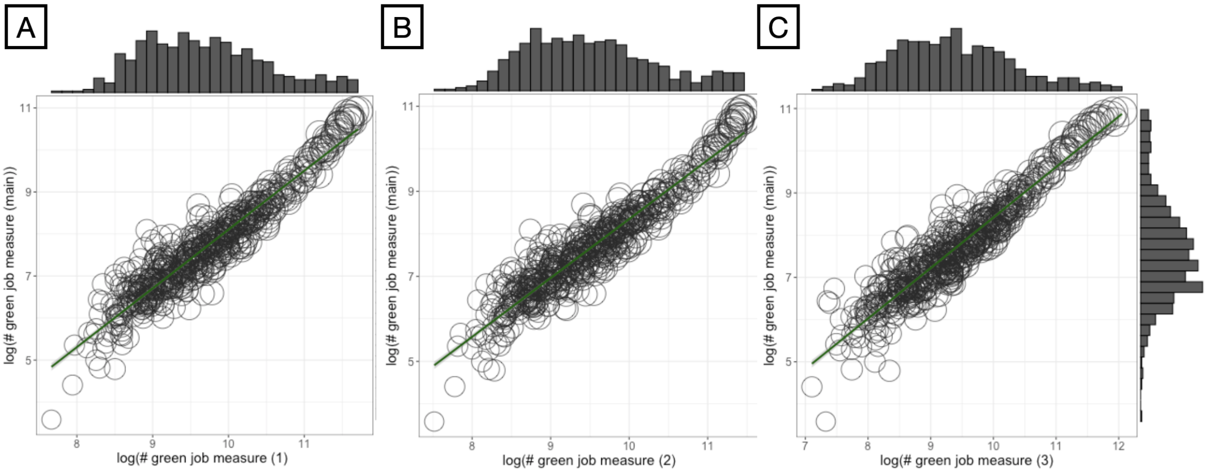

SI Figure 4: Comparison between different measures of green jobs. The scatter plots compare the measure of the logged number of green jobs used in the primary analysis (Renewable Energy Generation) with alternative measures of green jobs including the predicted number of employments **A** in occupations in the renewable energy sector according to the BLS projection for 2029 (25 occupations), **B** in the renewable energy sector (solar, wind, biomass, hydro) that is projected to grow either in the number or share in employment according to the BLS projection (21 occupations), **C** Green Increased Demand (GID) jobs defined by [2] beyond occupations in the renewable energy sector (52 occupations).

There are several alternative ways to identify green occupations. BLS provides the distribution of occupations (SOC 6 digit) across industries (NAICS 6 digit) at the national level as well as the projection for employment growth in 2029 by occupation by industry at the national level. Using the BLS projection along with the definition of green jobs provided by [2], we use the following alternative ways to estimate green jobs and show that these alternative estimates are highly correlated with the primary measure of green job estimates: (1) list of all occupations in the renewable energy sector in BLS projection for 2029 (Figure 4A, correlation coefficient $\rho = 0.941$), (2) occupations in the renewable energy sector (solar, wind, biomass, hydro) that is projected to grow either in the number or share in employment by BLS (Figure 4B, $\rho = 0.934$),(3) all the Green Increased Demand (GID) jobs defined, which are expected to experience growth in employment, by [2] beyond occupations in the renewable energy sector (Figure 4C, $\rho = 0.970$).

Lastly, we note that recent efforts try to identify green (energy) jobs based on online job posting sources such as Burning Glass [4]. While these offer valuable insights, they tend to overly represent white collar positions. Our focus lies on occupations that may be less visible in online searches.

2.2 Comparison with other measures of green occupations

2.2.1 Validating prediction for green job supply in 2029

We created predictions for green occupation employment in 2029 using historical data from the BLS on occupation employment distribution in MSAs and NMSAs in the US from 2005 to now, combined with Census data (population by age, gender, race, and education) and economic features (state-level

GDP per capita by sector). With this historical data, we train a model that, for each year, predicts an employment projection for 10 years into the future (e.g., predicts occupation employment in 2016 using data from 2006 and 2005). Since we aim to predict the employment projection in 2029 using the currently available data, we train the model to predict occupation employment using the data from 10 years lagged (i.e., we will apply the trained model to 2019 data to predict 2029 employment). We compare the performance of a random forest model against other types of models (OLS, Poisson, Lasso). We use ten-fold cross-validation to tune the hyper-parameters of the model.

We cannot directly evaluate the accuracy of our predictions for 2029 green employment. But we can use the historical employment data to train and test the accuracy of model predictions in a historical context. To avoid data leakage, we separated the training and test sets using the temporal cutoff by setting aside the 2019 employment distribution data as a test set and using the previous years to train the model. The best-performing model achieves $R^2 = 0.820$ when using pre-2019 data to predict 2019 employment outcomes.

Model	(1)	(2)	(3)	(4)	(5)	(6)	(7)	(8)	(9)
Estimator	OLS	Poisson	Lasso	Lasso	Lasso	RF	RF	RF	RF
Hyper-Parameter			alpha = 1	alpha = 0.1	alpha = 0.01	N_est= 1000 Max_depth= 3	N_est = 1000 Max_depth= 5	N_est= 1000 Max_depth= 7	N_est= 1000 Max_depth= 8
R^2	0.593	0.638	0.359	0.581	0.590	0.786	0.809	0.815	0.820
RMSE	0.854	0.805	1.072	0.866	0.857	0.620	0.586	0.576	0.572

SI Table 3: Comparing model performance when predicting 2019 green job employment.

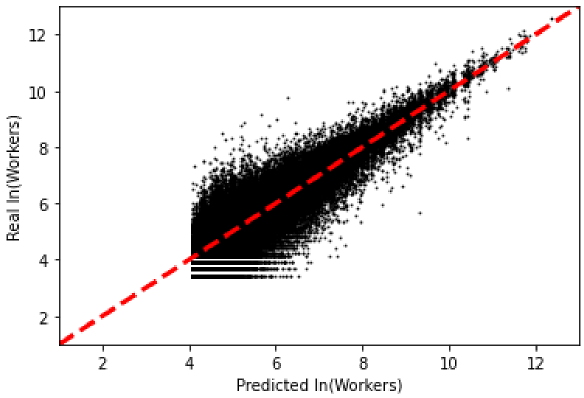

SI Figure 5: Validating the prediction for the number of green jobs. We compare (out-of-sample) predicted value for green jobs in 2019 (Predicted $ln(workers)$) and true value in 2019 (Real $ln(Workers)$). Red line refers to $y = x$. Prediction value is from model (9) in table 3: Random forest estimator with N_est = 1000, Max_depth = 8. Model achieved $R^2 = 0.820$, $RMSE = 0.572$

2.2.2 Comparison: Princeton Net-Zero America Project

The Princeton Net-Zero America Project (`https://netzeroamerica.princeton.edu`) analyzes five alternative pathways to the complete decarbonization of the US economy compared to a baseline scenario from the US Energy Information Administration (EIA) [5]. They compute state-level estimates of employment in various renewable energy sectors disaggregated by energy source (i.e. solar, wind, biomass) (see

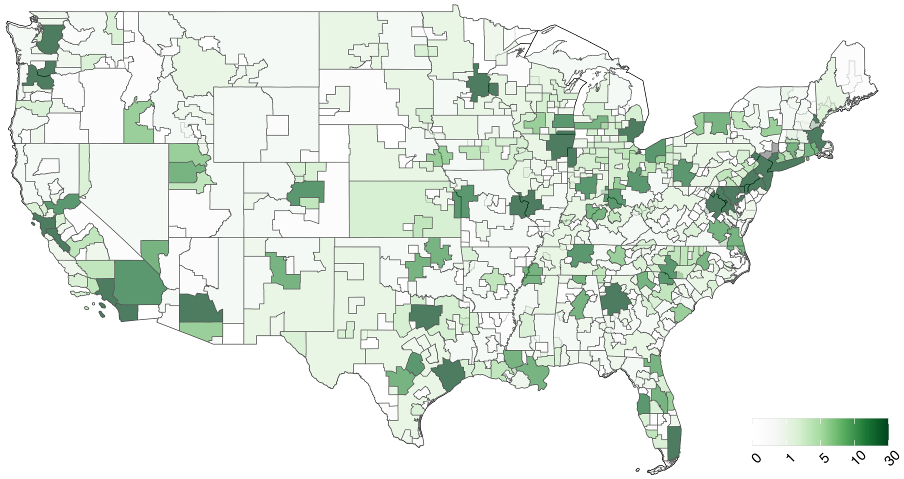

SI Figure 6: Geographical distribution of the predicted number of green jobs in 2029. Prediction for the number of green jobs in 2029 is generated based on model (9) from Table 3. The reported value on the map is logged. Maps were made using the sf package in R (Pebesma E (2018). "Simple Features for R: Standardized Support for Spatial Vector Data." The R Journal, 10(1), 439–446.–CC-BY Attribution 4.0).

Annex R, `https://netzeroamerica.princeton.edu/img/NZA%20Annex%20R%20-%20Labor%20modeling%20methodology.pdf`.). They do so by estimating how employment responds to changes in production (e.g. how employment in oil extraction changes when oil production increases). They can then use these parameters to generate predictions based on energy production in each of their scenarios.

We compare our employment estimates and our results with the data provided by the Net-Zero Project in Figure 7. To do so, we aggregate our predicted employment for green occupations in 2029 by state (note our estimates are made at the MSA/NMSA level). We then compare our aggregated employment predictions with the estimates from Net-Zero America project. Among various scenarios in Net-Zero America, we use the scenario "References," which predicts the number of green occupations in the renewable energy sector assuming no major policy intervention, and the same (low) projected oil and gas prices.

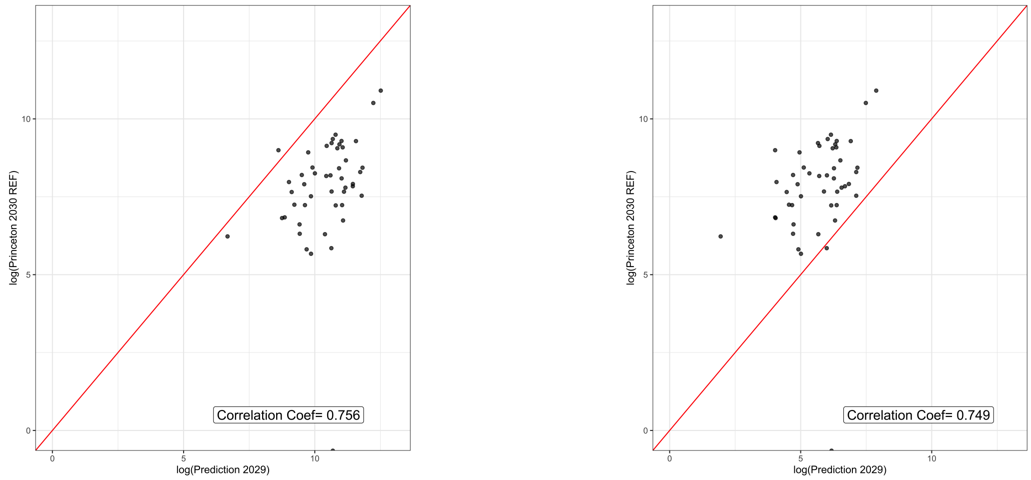

(a) Net-Zero Project - Green Employment (b) Net-Zero Project - Green Employment (weighted)

SI Figure 7: Comparing different estimates of green employment predictions

Figure 8 shows the prediction of the number of fossil-fuel workers who are likely to transition to green occupations given the predicted spatial distribution of green jobs. Using the regression model (4) from the Figure 1B in the main text, along with the state-level prediction of green jobs in various scenarios, we predict the number of extraction workers who will transition to green jobs. The number of extraction workers is summed up at the state level as well. Geospatial distance is calculated based on the centroid of each state using the haversine formula.

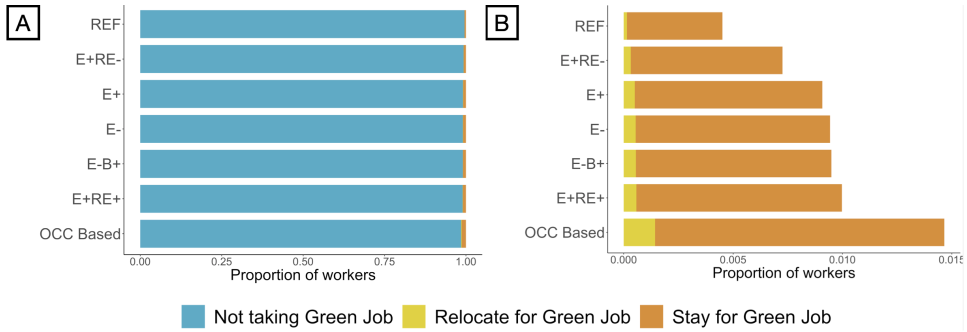

SI Figure 8: Prediction at the state level for transition of fossil fuel workers to green jobs. We compare the predicted values of the share of fossil fuel workers transitioning to green jobs using occupatiol based estimates of green jobs (OCC Based) and other estimates of green jobs from Princeton Net-Zero project under different scenarios.

3 Skill similarities between industries and occupations

3.1 Important skills for fossil-fuel workers

We identify the workplace skills, knowledge, and abilities that are important to fossil fuel extraction workers by averaging the importance of skills across 11 extraction occupations provided by the O*NET

database weighted by the size of employment in the fossil fuel sector in 2019. The list of occupations is in Table 1. We identify (1) the skills that have the highest value for O*NET skill values for fossil fuel (extraction) occupations (Table 4), which ranges 0 to 1 where 1 being the most important, and (2) the skills that are relatively more important to fossil fuel (extraction) occupations than for the average (Table 5). To identify the relative importance of skills for extraction workers (2), we compute the location quotient (LQ) of skills by dividing the average skill scores of extraction occupations by the average of skill values across every occupation. If the LQ of a skill is higher than 1, this skill is more important to fossil fuel (extraction) workers than the average occupation.

O*NET Skill	Description	Value	LQ
Realistic	Designing, building, or repairing of equipment, engaging in physical activity	1.00	1.57
Handling and Moving Objects	Using hands and arms in handling, installing, positioning, and moving materials	0.83	1.64
Performing General Physical Activities	Performing general physical activities	0.77	1.69
Controlling Machines/Processes	Using control mechanisms or physical activity to operate machines (excluding computers, vehicles)	0.77	1.82
Inspecting Equipment/Structures/Material	Inspecting equipment, structures, or materials to identify the cause of errors	0.76	1.57
Monitor Processes/Materials/Surroundings	Monitoring and reviewing information from materials, events, or the environment	0.69	1.15
Communicating w Supervisors/Peers/Subordinates	Providing information to supervisors, co-workers, and subordinates	0.69	1.03
Identifying Objects/Actions/Events	Identifying information by categorizing, and recognizing differences or similarities	0.67	1.05
Mechanical	Having knowledge of machines and tools, including their designs, uses, repair, maintenance.	0.66	1.91
Repairing/Maintaining Mechanical Equipment	Servicing, repairing, adjusting, and testing machines devices, and moving parts	0.64	2.16

SI Table 4: Top 10 most important skills for fossil fuel (extraction) workers according to skill value. Value refers to the O*NET skill score that indicates the importance of each skill for extraction workers. This ranges from 0 to 1, where 1 refers to the most important skill. The above 10 skills are skills with the highest O*NET values for fossil fuel (extraction) occupations. Location quotient (LQ) measures the relative importance of each skill for extraction workers. If LQ of a skill is higher than 1, this skill is more important to fossil fuel (extraction) occupations than the average.

O*NET Skill	Description	Value	LQ
Sound Localization	to tell the direction from which a sound originated	0.32	3.24
Night Vision	to see under low-light conditions	0.27	3.15
Repairing	to repair machines or systems using the needed tools	0.47	2.88
Glare Sensitivity	to see objects in the presence of a glare or bright lighting.	0.33	2.86
Peripheral Vision	to see objects or movement of objects to one's side	0.27	2.77
Equipment Maintenance	to perform routine maintenance on equipment	0.47	2.76
Speed of Limb Movement	to quickly move the arms and legs	0.36	2.57
Spatial Orientation	to know your location in relation to the environment	0.34	2.52
Installation	to install equipment, machines, wiring, or programs to meet specifications	0.14	2.50
Gross Body Equilibrium	to keep/regain your body balance or stay upright in an unstable position.	0.42	2.44

SI Table 5: Top 10 skills with relative importance for fossil fuel (extraction) workers according to location quotient. The above 10 skills are skills with the highest location quotient (LQ) for fossil-fuel (extraction) workers. LQ measures the relative importance of each skill for extraction workers in comparison to its importance for other occupations. If LQ of a skill is higher than 1, this skill is more important to fossil fuel (extraction) workers than the average occupation.

3.2 Important skills for green jobs

Similarly, We identify important skills for green occupations in renewable energy generation sector by taking an average of the importance of skills, knowledge, and abilities across these occupations (provided by O*NET) weighted by the size of employment in 2019. The list of occupations is in Table 1. We identify (1) the skills that have the highest value for O*NET skill values for green occupations (table 6), which ranges 0 to 1 where 1 being the most important, and (2) the skills that are relatively more important to green occupations than for the average (Table 7) using the location quotient (LQ) by dividing skill scores of green occupation by the average of skill values across every occupation.

O*NET Skill	Description	Value	LQ
Realistic	Designing, building, or repairing of equipment, engaging in physical activity	0.83	1.31
Making Decisions and Solving Problems	Analyzing information and evaluating results	0.74	1.11
Getting Information	Observing, receiving, and otherwise obtaining information from all relevant sources	0.73	1.06
Communicating with Supervisors, Peers, or Subordinates	Providing information to co-workers	0.72	1.08
Updating/Using Relevant Knowledge	Keeping up-to-date technically and applying new knowledge to your job	0.71	1.09
Identifying Objects, Actions, Events	Identifying information by categorizing, estimating, recognizing differences or similarities	0.71	1.11
Monitor Processes, Materials, or Surroundings	Monitoring and reviewing information from materials, events, or the environment	0.71	1.18
Evaluating Information to Determine Compliance with Standards	Using information and judgment to determine whether events or processes comply with laws or standards	0.68	1.22
Processing Information	Compiling, coding, categorizing, calculating, tabulating, auditing, or verifying information or data	0.68	1.14
Inspecting Equipment, Structures, Material	Inspecting equipment, structures, or materials to identify the cause of errors	0.67	1.37

SI Table 6: Top 10 most important skills for green jobs in the renewable energy sector according to skill value. Value refers to the O*NET based score that indicates the importance of each skill for green occupations in renewable energy generation sector. This ranges from 0 to 1, where 1 refers to the most important skill. The above 10 skills are skills with the highest O*NET values for green occupations in renewable energy generation sector.

O*NET Skill	Description	Value	LQ
Physics	Having knowledge of physical principles	0.51	2.18
Repairing	Repairing machines using the needed tools	0.32	1.95
Engineering and Technology	Having knowledge of the design, development, and application of technology for specific purposes	0.60	1.92
Drafting, Laying Out, Specifying Technical Devices	Providing documentation, detailed instructions, to tell others about how devices, parts, equipment are to be fabricated	0.46	1.86
Equipment Maintenance	Performing routine maintenance on equipment and determining when and what kind of maintenance is needed.	0.30	1.78
Troubleshooting	Determining causes of operating errors and solutions	0.43	1.78
Science	Using scientific rules and methods to solve problems	0.36	1.72
Repairing and Maintaining Electronic Equipment	Servicing, repairing, calibrating, regulating, fine-tuning, or testing machines, devices, and equipment	0.45	1.71
Design	Having knowledge of design techniques, tools, and principles involved in production of precision technical plans.	0.48	1.69
Mechanical	Having knowledge of machines and tools, including their designs, uses, repair, and maintenance.	0.56	1.61

SI Table 7: Top 10 skills with relative importance for green occupations in the renewable energy sector according to location quotient. The above 10 skills are skills with the highest location quotient (LQ) for green occupations.

3.3 Skill similarities across industries

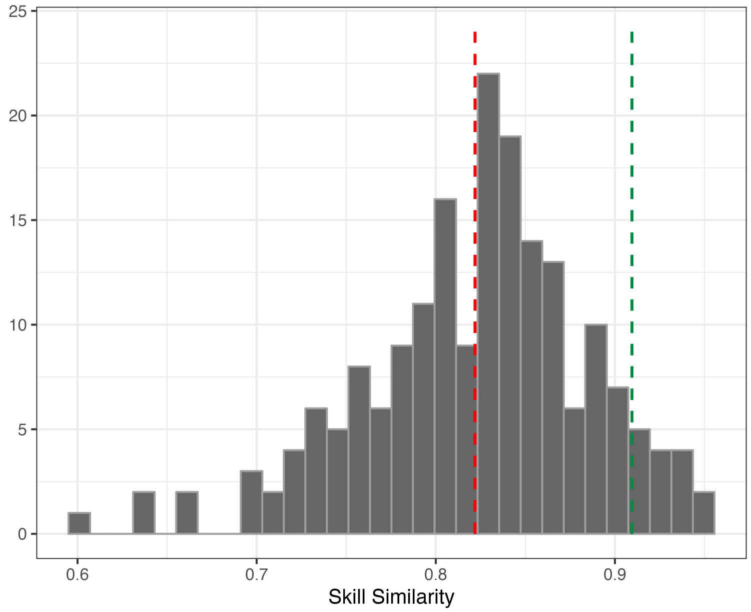

SI Figure 9: Distribution of pair-wise skill similarities across all the industries (NAICS 2 digit). The mean of pair-wise skill similarities = 0.82 (red line) and the skill similarity score between fossil-fuel industry and green jobs = 0.91 (green line).

Industry 1	Industry 2	Skill Similarity Score
Professional, scientific, and technical services	Management of companies and enterprises	0.96
Mining, quarrying, and oil and gas extraction	Construction	0.94
Wholesale trade	Real estate and rental and leasing	0.94
Information	Professional, scientific, and technical services	0.94
Real estate and rental and leasing	Other services (except public administration)	0.93
Wholesale trade	Other services (except public administration)	0.93
Information	Management of companies and enterprises	0.93
Finance and insurance	Management of companies and enterprises	0.93
Arts, entertainment, and recreation	Other services (except public administration)	0.92
Retail trade	Arts, entertainment, and recreation	0.92

SI Table 8: 10 pairs of industries with the most similar skill requirements (NAICS).

NAICS	Description	Similarity
23	Construction	0.94
22	Utilities	0.89
48-49	Transportation and Warehousing	0.88
81	Other Services (except Public Administration)	0.85
31-33	Manufacturing	0.85
53	Real Estate and Rental and Leasing	0.84
42	Wholesale Trade	0.84
56	Administrative and Support and Waste Management and Remediation Services	0.83
71	Arts, Entertainment, and Recreation	0.81
62	Health Care and Social Assistance	0.80
44-45	Retail Trade	0.80
11	Agriculture, Forestry, Fishing and Hunting	0.79
51	Information	0.79
72	Accommodation and Food Services	0.79
54	Professional, Scientific, and Technical Services	0.76
55	Management of Companies and Enterprises	0.76
61	Educational Services	0.74
52	Finance and Insurance	0.72

SI Table 9: Skill similarities between Fossil-fuel workers and other industries (NAICS)

4 Modeling Fossil Fuel Workers Transitions to Other Industries

To model the transition of fossil-fuel workers, we use the Job-to-Job Origin-Destination (J2JOD) data from the US Census Bureau, which provides information regarding the number of workers flowing from one industry-location to another every quarter. Information on location and industry is available at the metropolitan area, and NAICS 2-digit level, respectively.

Figure 10A shows the share of fossil fuel workers taking new jobs (in any industry) as a function of distance from their point of origin. Each line represents the mean value for a metropolitan area. On average, slightly more than 20% of fossil fuel workers who switch jobs stay in the same metropolitan area. Furthermore—and consistent with studies on labor market mobility—fossil fuel workers seldom move far to take new jobs. The share of fossil fuel workers who move further than 50 miles (∼80km) to start a new job is extremely small (Figure 10A). This suggests a high degree of sedentary for a large proportion of fossil fuel workers.

Next, we repeat the same exercise but consider skill similarity. We ask: what is the proportion of fossil fuel workers who take new jobs with identical skill requirements, and how does the likelihood of taking a new job decline as the set of skills required in the new job becomes increasingly different? To answer these questions, we use O*NET data to identify the skill set needed by the average worker in the fossil fuel sector. We then compute the difference in skill sets between their old (fossil fuel) job and their new one. We measure this as the Jaccard similarity score, which can informally be interpreted as the percentage of similarity between two vectors (here: of O*NET skills, knowledge, and abilities). Figure 10B shows that fossil fuel workers are most likely to take jobs with $skillsim(f, i) \geq 0.9$, suggesting that whenever they change jobs, it is to take a new position that has very similar skill requirements.

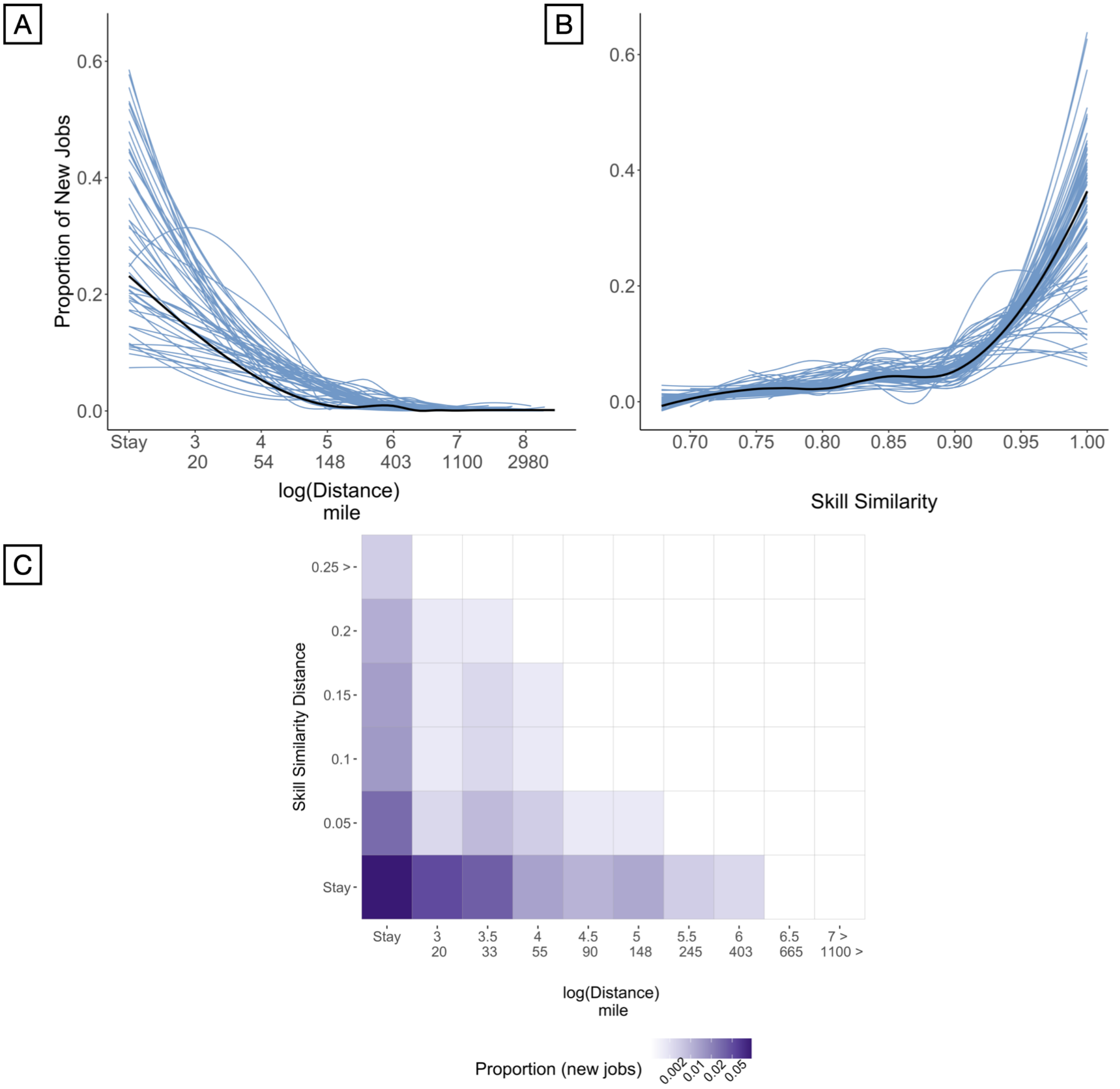

SI Figure 10: Fossil fuel workers are willing to move (up to about 50 miles) or switch to jobs with similar skills, but not both. According to the J2JOD data provided by the US Census Bureau, **A** share of fossil fuel workers taking jobs in different industry as a function of the geographic distance between their previous and their new occupation. Each blue line represents a metropolitan area, and the black line represents the average across metropolitan areas. **B** share of fossil fuel workers taking jobs in different industry as a function of skill similarity between their old and their new job. **C** share of fossil fuel workers taking jobs in different industry as a function of both geographical distance and skill similarity between industries.

We estimate the following model's predictive performance using 10-fold cross-validation:

$$\begin{aligned}\log\left(\text{Transition}_{f,m,i,m'}\right) \sim \beta_0 + \beta_1 \log\left(\text{Distance}\right)_{m,m'} + \beta_2 skillsim(f,i) \\ + \beta_3 Employment_{f,m} + \beta_4 Employment_{i,m'}\end{aligned} \tag{1}$$

where m and m' are regions (i.e., metropolitan statistical areas or non-metropolitan statistical areas), f represents fossil fuel workers, and i is target industry/sector (i.e., two-digit NAICS code). Employment statistics come from the Occupation Employment and Wage Statistics from the BLS.

We created a prediction for the distribution of green occupation employment in 2029 using historical data from the BLS on occupation employment distribution in metropolitan (MSA) and non-metropolitan (NMSA) areas in the US from 2005 to now. With this historical data, we train a random forest regression model to predict future employment distributions by region. In addition, we include other demographic data from US Census Bureau's American Community Survey (ACS) including the population by age, gender, race, education in each area, and economic features (state-level GDP per capita by sector). Since we aim to predict employment in 2029 using the currently available data, we train the model to predict occupational employment using the data from 10 years lagged. We compare the performance of random forest against other classifiers (OLS, Lasso) according to 10-fold cross-validation while tuning hyperparameters of the models. Whereas we cannot directly evaluate the accuracy of our prediction for the distribution of green jobs in 2029, we could use the historical employment data to train and test the accuracy of predictions. To avoid data leakage, we separated the training set and test set using the temporal cutoff. We set aside the 2019 employment distribution data as a test set while training the model with the previous years.

4.1 Transition under different scenarios

In Figure 4A of the main text, we explore multiple scenarios for fossil fuel workers transitioning to either (1) occupations in other sectors (left side of the figure) or (2) green occupations (right side of the figure).

4.2 Transition to non-green sectors

For the scenarios where fossil fuel workers transition to the existing industries, we consider six different sectors: Construction, Manufacturing, Transportation and Warehousing, Education Services, Science and Technology, and Finance and Insurance. These industries include those with the three highest and lowest levels of skill similarity with fossil fuel sector occupations (see SI section 3.3).

To estimate the fossil fuel workers' transition rates to each industry, we first identify the core occupations of each industry (defined by 2-digit NAICS codes). Then, we find the predicted number of jobs in each occupation and industry in 2029 for both metro and non-metropolitan areas using the same prediction method (see SI section 2.2). Lastly, using these prediction along with the regression model from Figure 1B, model (5) in the main text, we project the potential transition to these industries.

We identify the core occupations using national employment projection data provided by the Bureau of Labor Statistics (BLS), which reports the number of workers by occupation (i.e., 6-digit Standard Occupation Classification (SOC) code) and industry (i.e., NAICS code) at the national level. From this data, we determine the core workforce of each occupation by calculating the conditional probability of working in industry i given having occupation o ($Pr(\text{Working for Industry}_i|\text{Having Occupation}_o)$). Using this information, we then identify the core occupation of each industry. For example, we identified 82 occupations in the manufacturing industry with $Pr(\text{Working for Manufacturing}|\text{Having Occupation}_o) > 0.6$, which comprises 45% of the entire manufacturing workforce.

Additionally, we also identified the occupations that are most likely for fossil fuel workers to transition to, using the SOC major and minor group code, instead of industry level with the NAICS code.

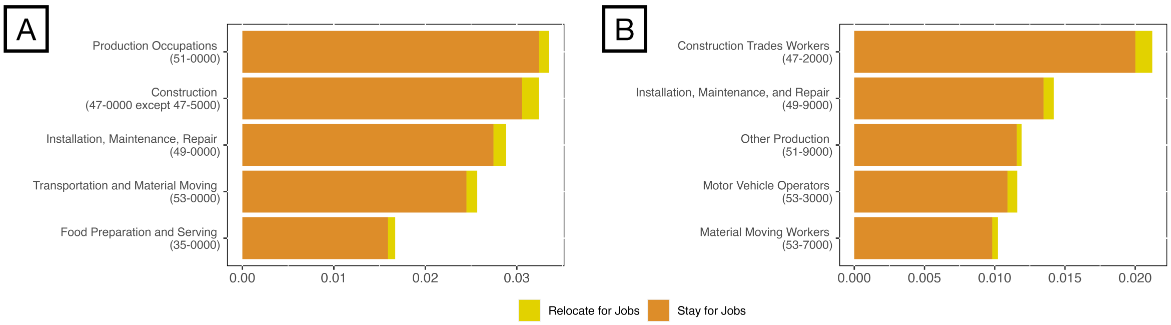

SI Figure 11: 5 major (SOC 2 digit code) and minor occupation groups (SOC 3 digit code) with the highest transition rates of fossil-fuel extraction workers

4.3 Transition to green jobs under different jobs supply scenario

To estimate the effect of potential green job growth on fossil fuel workers' transition to green occupations, we simulate 6 different scenarios with the combination of (1) different total number of additional jobs and (2) whether the distribution of job is geographically targeted toward fossil fuel labor intensive regions or not.

For non-targeted scenarios, we increase the number of the fossil fuel jobs with the equal rate for every metro and non metropolitan areas from our original predicted number of green jobs. For geo-targeted scenarios, we distribute the green jobs proportional to the share of fossil fuel workers across metro and non metropolitan areas. Across scenarios, the number of each green job (i) in each region m evolves following:

$$GOCC_{i,m} + (\#\text{ of Jobs created}) \times \frac{GOCC_{i,m}}{\sum_{i,m} GOCC_{i,m}} \qquad \text{(Non-Targeted)}$$

$$GOCC_{i,m} + (\#\text{ of Jobs created}) \times \frac{GOCC_{i,m}}{\sum_{i} GOCC_{i,m}} \times \frac{\sum_{i} FOCC_{i,m}}{\sum_{i,m} FOCC_{i,m}} \qquad \text{(Geo-Targeted)}$$

where $GOCC_{i,m}$ as the predicted number of green job i in area m in the baseline scenario (without considering any policy interventions), $FOCC_{i,m}$ as the number of fossil-fuel extraction job i in area m in 2019. In our analyses, we vary "# of Jobs created" by 1 million (1M), 5 million (5M), and 10 million (10M). Across these six different scenarios, we assume that the the proportion of different green occupation within the region are the same with our baseline scenario prediction for 2029 ($\frac{G{-}OCC_{i,m}}{\sum_i G{-}OCC_{i,m}}$). In other words, we do not take into account specific occupation based targeting. With these different scenarios, we use our transition model to predict the proportion of fossil fuel workers in 2019 that will be able to transition.

Figure 12 shows the different spatial distribution of green jobs by whether policy interventions to create green jobs are geo-targeted (Fig 12 B) or not (Fig 12 A).

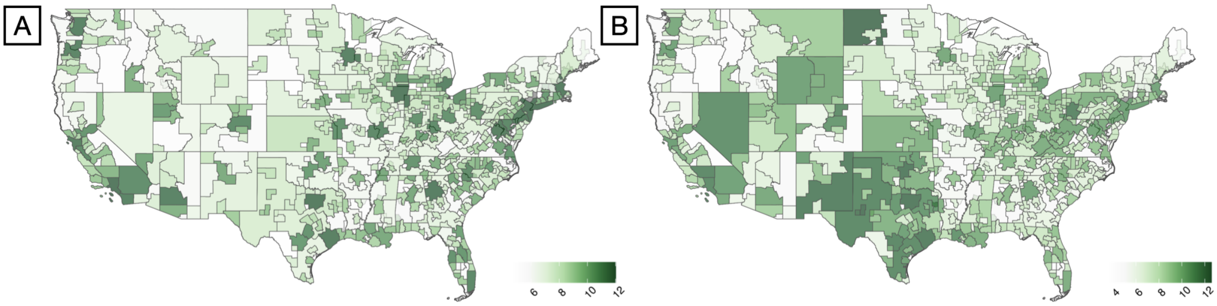

SI Figure 12: Distribution of the logged number of green jobs under **A** scenario where 5 million additional green jobs distributed proportional to the current distribution of green jobs (Non-Targeted (5M)), **B** scenario where 5 million additional green jobs distributed proportional to the current distribution of fossil fuel extraction workers (Geo-Targeted (5M)). Maps were made using the sf package in R (Pebesma E (2018). "Simple Features for R: Standardized Support for Spatial Vector Data." The R Journal, 10(1), 439–446.–CC-BY Attribution 4.0).

4.4 Transition to different types of green jobs

We examine potential transition from fossil-fuel extraction jobs to other types of green jobs. While we focus on green jobs that are related to the renewable energy generation for our main analyses, there are different types of green occupations defined by [2] (see SI Section 2.1 for detailed explanation on different types of green jobs). Using the identical approach with the SI Section 4.1, we project the transition rates of fossil-fuel extraction workers to different types of green jobs.

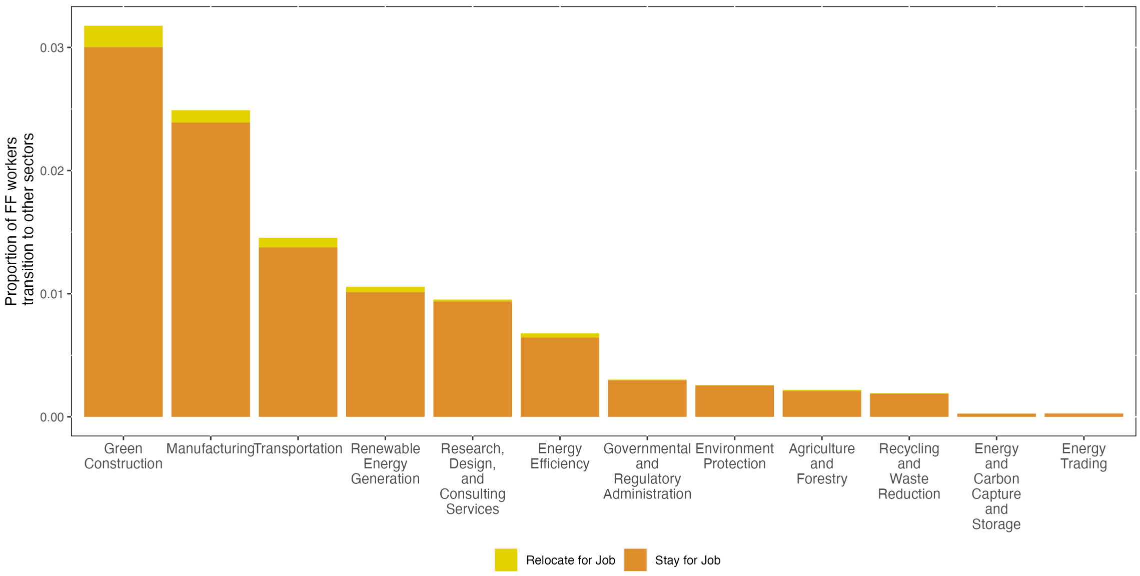

SI Figure 13: Transition to different types of green jobs

5 Fossil Fuel Workers and Green Energy Plants

Another way to examine whether fossil fuel workers are located near renewable energy sector jobs is to compare the current location of fossil fuel workers and green energy plants. We use the geo-coded data on the location of power plants by different renewable energy sources (solar, hydro, wind, biomass), provided by U.S. Energy Information Administration.

	Dependent variable:							
	# Solar		# Hydro		# Biomass		# Wind	
	(1)	(2)	(3)	(4)	(5)	(6)	(7)	(8)
log(Extraction Workers)	−0.06***		−0.01		−0.06***		0.18***	
	(0.01)		(0.01)		(0.01)		(0.01)	
log(FF Workers)		−0.12***		0.07***		−0.05***		0.19***
		(0.01)		(0.01)		(0.02)		(0.01)
log(Labor Force)	0.70***	0.80***	0.20***	0.14***	0.56***	0.59***	−0.005	−0.06**
	(0.01)	(0.01)	(0.02)	(0.02)	(0.03)	(0.03)	(0.03)	(0.03)
Pseudo R^2	0.33	0.35	0.02	0.03	0.32	0.32	0.09	0.06
Observations	510	510	510	510	510	510	510	510
Note:								*p<0.1; **p<0.05; ***p<0.01

SI Table 10: Location of the US fossil-fuel workers and green energy plants using Poisson regression.

Supplementary References

[1] USDA. County-level oil and gas production in the u.s. Database, 2014.

[2] Erich C. Dierdorff, Jennifer J. Norton, Donald W. Drewes, Christina M. Kroustalis, David Rivkin, and Phil Lewis. Greening of the world of work: Implications for o*net-soc and new and emerging occupations. U.S. Department of Labor Report, February 2009.

[3] F. Vona, F. Biagi, and A. Bitat. Labour markets and the green transition: a practitioner's guide to the task-based approach. Publications Office of the European Union, 2021.

[4] E Mark Curtis and Ioana Marinescu. Green energy jobs in the us: What are they, and where are they? NBER Working Paper 30332, 2022.

[5] Eric Larson, Chris Greig, Jesse Jenkins, Erin Mayfield, Andrew Pascale, Chuan Zhang, Joshua Drossman, Robert Williams, Steve Pacala, Robert Socolow, et al. Net-zero america: Potential pathways, infrastructure, and impacts, interim report. Technical report, Princeton University, 2020.
